# Population genetic structure and connectivity of *Enhalus acoroides* along the Southern Vietnamese coastline

**DOI:** 10.3389/fpls.2026.1953986

**Published:** 2026-10-01

**Authors:** Xuan-Vy Nguyen, Thi Thuy Hang Phan, Nhu-Thuy Nguyen-Nhat, Xuan-Thuy Nguyen, Trung-Hieu Nguyen, My-Ngan Nguyen, Viet-Ha Dao, Jutta Papenbrock

**Affiliations:** 1 Institute of Oceanography, Vietnam Academy of Science and Technology, Nha Trang, Vietnam; 2 University of Sciences, Hue University, Hue, Vietnam; 3 Institute of Botany, Leibniz University Hannover, Hannover, Germany

**Keywords:** conservation, Enhalus, genetic diversity, migration rates, Vietnam

## Abstract

**Introduction:**

Seagrass meadows have undergone substantial declines in Vietnam over recent decades owing to habitat loss, climate change, nutrient enrichment, and other anthropogenic disturbances. *Enhalus acoroides*, a dominant large tropical seagrass, has experienced marked reductions in distribution and abundance, yet information on its population genetic structure remains limited.

**Methods:**

We analyzed genetic diversity, population structure, and contemporary connectivity using 12 microsatellite loci genotyped from 154 multilocus genotypes collected from ten populations distributed across the Eastern waters (central Vietnam, South China Sea) and Western waters (An Giang coast, Gulf of Thailand), with distances among sampling locations ranging from 20 to 1,250 km.

**Results:**

High clonal diversity (R = 0.77–1.00) indicated that populations were maintained predominantly through sexual reproduction. Genetic diversity varied among populations, with the highest allelic richness observed in Van Phong Bay and Cam Ranh Bay, whereas Cu Mong Lagoon exhibited the lowest diversity. Population genetic analyses revealed significant genetic differentiation, restricted connectivity between the Eastern and Western waters, and a weak but significant pattern of isolation by distance. Nha Trang Bay was highly differentiated from all other populations and received very limited contemporary gene flow, suggesting the influence of habitat-associated isolation. Based on migration rates and relative genetic contribution, Van Phong Bay, Cam Ranh Bay, and Hai Tac Archipelago were identified as candidate source populations for conservation and restoration.

**Discussion:**

These findings provide a comprehensive assessment of the population genetic structure of *E. acoroides* in Vietnamese waters and provide a scientific basis for conserving and restoring tropical seagrass ecosystems dominated by *E. acoroides*.

## 1 Introduction

Seagrasses are marine angiosperms that complete their life cycles in brackish and marine environments and provide essential ecosystem services, including sediment stabilization, water-quality improvement, biodiversity support, and blue carbon storage (Fourqurean et al., 2012; do Amaral Camara Lima et al., 2023; Gomis et al., 2025). Despite their ecological and economic importance, seagrass meadows are declining worldwide due to multiple interacting stressors, including coastal development, eutrophication, pollution, declining water quality, and climate change (Waycott et al., 2009; Duarte et al., 2025). This widespread degradation can reduce habitat quality and ecosystem services, cause biodiversity loss, and alter seagrass community composition (de Fouw et al., 2024; Patriquin et al., 2024). Consequently, seagrass restoration has become a global conservation priority, particularly for biodiversity conservation, blue carbon, and climate mitigation (Duarte et al., 2025; Krause et al., 2025). Although restoration outcomes have improved in many regions, success varies among species, habitats, and restoration approaches (Kendrick et al., 2025; Thorhaug et al., 2025). Importantly, genetic diversity and connectivity among populations can influence the availability of genetically diverse donor material and may contribute to the resilience and long-term persistence of restored meadows (Jackson et al., 2021). At the same time, habitat loss and fragmentation can disrupt reproductive and dispersal processes, potentially reducing gene flow and genetic connectivity among seagrass meadows (Hernawan et al., 2023). Understanding the factors that maintain or constrain genetic connectivity is therefore important for assessing population resilience and informing effective conservation and restoration strategies (Kendrick et al., 2012; Kendrick et al., 2025).

Genetic diversity is a fundamental component of biodiversity that contributes to the persistence, adaptive capacity, and evolutionary potential of natural populations (Hughes et al., 2008; Reusch et al., 2005). In seagrasses, maintaining high genetic diversity and connectivity may enhance resilience to environmental change and reduce the potential effects of inbreeding and genetic drift. Therefore, assessing genetic diversity and population connectivity is important for identifying conservation units and selecting appropriate donor populations for restoration (Jahnke et al., 2020; Pazzaglia et al., 2021; Unsworth et al., 2025). Microsatellite (SSR) markers have been widely used to assess genetic diversity, clonality, population structure, and gene flow in seagrasses because of their high polymorphism and codominant inheritance (Ben-Ari and Lavi, 2012). Previous SSR-based studies have revealed substantial variation in genetic structure and connectivity among seagrass species, highlighting the importance of species-specific reproductive, dispersal, and environmental processes in shaping population genetic patterns (Nguyen et al., 2022a; Arriesgado et al., 2023; Unsworth et al., 2025).

Understanding the reproductive and dispersal characteristics of *Enhalus acoroides* is important for interpreting its population connectivity and genetic structure, as sexual reproduction, pollen and seed dispersal, and oceanographic processes can influence gene flow among populations (Nakajima et al., 2014; Yu et al., 2019). *Enhalus acoroides* is a dioecious seagrass that reproduces sexually through hydrophilous pollination at the water surface, followed by fruit and seed development (Lacap et al., 2002). The dispersal of reproductive propagules, including pollen, fruits, and seeds, may be influenced by seawater movement, with local oceanographic conditions and hydrodynamic connectivity potentially affecting gene flow and population genetic structure among habitats (Rollón et al., 2003; Yu et al., 2019). Such dispersal processes may facilitate gene flow among geographically connected *E. acoroides* meadows, whereas greater geographic distances, habitat discontinuity, and oceanographic barriers may limit connectivity and contribute to genetic differentiation among populations (Dierick et al., 2021). Consequently, variation in reproductive success, propagule dispersal, and hydrodynamic connectivity among meadows may contribute to the spatial genetic structure of *E. acoroides* populations (Yu et al., 2018). Assessing genetic connectivity can therefore help clarify the processes shaping genetic diversity and identify populations that may warrant priority for conservation and restoration.

Vietnam is recognized as one of the centers of seagrass diversity in Southeast Asia, supporting approximately 15, 636 ha of seagrass distributed along more than 3, 260 km of coastline (Nguyen-Nhat et al., 2024). However, seagrass coverage has declined by approximately 46.5% since 1990 (Nguyen et al., 2022a), and the dominant species, *Enhalus acoroides*, has experienced substantial habitat loss because of aquaculture expansion, coastal development, land reclamation, and extreme weather events (Chen et al., 2016; Vo et al., 2020; Nguyen et al., 2021). The first genetic study of *E. acoroides* in Vietnam was conducted by Nguyen et al. (2015), who applied DNA barcoding to confirm species identification. Previous studies on *E. acoroides* showed that meadows along the coastline of Khanh Hoa Province exhibit reduced genetic diversity, as well as pronounced genetic differentiation between lagoon sites and open-sea populations (Nguyen and Papenbrock, 2019). Dierick et al. (2021) described clonal richness, genetic diversity, and dispersal within and among populations in four lagoons based on analyses of eight *E. acoroides* populations along the South-Central Coast of Vietnam using 11 polymorphic microsatellite loci. The authors demonstrated that large, persistent genets increase the resilience of *E. acoroides* meadows under high disturbance regimes and revealed significant genetic differentiation among lagoons, suggesting that these systems act as barriers to gene flow. The genetic diversity and population structure of *E. acoroides* are well documented in several locations in Asia such as Indo-Malay Archipelago (Putra et al., 2018), population among the Philippines, Japan, and China (Nakajima et al., 2014), Hainan, China (Yu et al., 2018, Yu et al., 2019). A better understanding of genetic connectivity between these regions may help inform the identification of conservation priorities and the selection of genetically appropriate source populations for seagrass restoration. However, although previous studies have included *E. acoroides* populations from central Vietnam, genetic connectivity between populations in the South China Sea and the Gulf of Thailand remains comparatively poorly understood. To address this gap, we examined *E. acoroides* populations from 10 sites distributed across two major marine regions of Vietnam: the eastern waters, comprising the central and southern coasts of mainland Vietnam, and the western waters, comprising the An Giang coast and Phu Quoc Island in the Gulf of Thailand. The sampling sites were separated by approximately 20–1, 250 km, providing a broad geographic framework for assessing population genetic structure and connectivity between these two regions.

This led to the following hypotheses: (*i*) populations separated by greater geographic distances would exhibit greater genetic differentiation, consistent with an isolation-by-distance pattern; (*ii*) populations that are geographically proximate and oceanographically connected would exhibit greater genetic connectivity than those separated by greater geographic distances or major oceanographic barriers; and (*iii*) differences in geographic and oceanographic settings between the eastern and western waters of the Vietnamese coast would contribute to detectable genetic structuring among populations.

## 2 Materials and methods

### 2.1 Sampling sites and collection

A total of 196 samples of the seagrass species *E. acoroides* were collected from six seagrass beds in the eastern waters (South China Sea): Cu Mong Lagoon (CM, site 1), Xuan Tu (XT, site 2), Hon Khoi (HK, site 3), Nha Trang Bay (NT, site 4), Cam Ranh Bay (CR, site 5), and Ninh Hai (NH, site 6); and four seagrass beds in the western waters: Kien Luong (KL, site 7), Ba Lua Archipelago (BL, site 8), Hai Tac Archipelago (HT, site 9), and Phu Quoc Island (PQ, site 10) (**Figure 1**). The four western populations (sites 7–10) are located along the coast of An Giang Province, formerly known as Kien Giang Province. Sampling at sites 1, 2, 3, 5, and 6 was conducted between March and April 2024, while the remaining sites were sampled in May 2025. Because the sampling periods broadly corresponded to the eastern and western geographic groupings examined in the population genetic analyses, the two regions were not sampled in the same year; however, given the long-lived and predominantly clonal nature of *E. acoroides*, this one-year difference is unlikely to have substantially influenced the observed genetic structure. Distances among sampling locations ranged from 20 to 1, 250 km. Within the study sites in the South China Sea, western border currents varied between winter and summer. During winter, strong currents flow southwestward along the South China shelf toward the coast of north-central Vietnam, then continue southward along the coasts of central and southern Vietnam, before partially exiting the South China shelf through the Karimata Strait. During the summer, the current flows north to about 11^°^N, after which it roughly splits into three branches. Two of the current branches turn eastward and south-east ward, while one flows northward (Fang et al., 2012). Water exchange between the Gulf of Thailand and the South China Sea is relatively weak (Mau et al., 2017).

**Figure 1:**
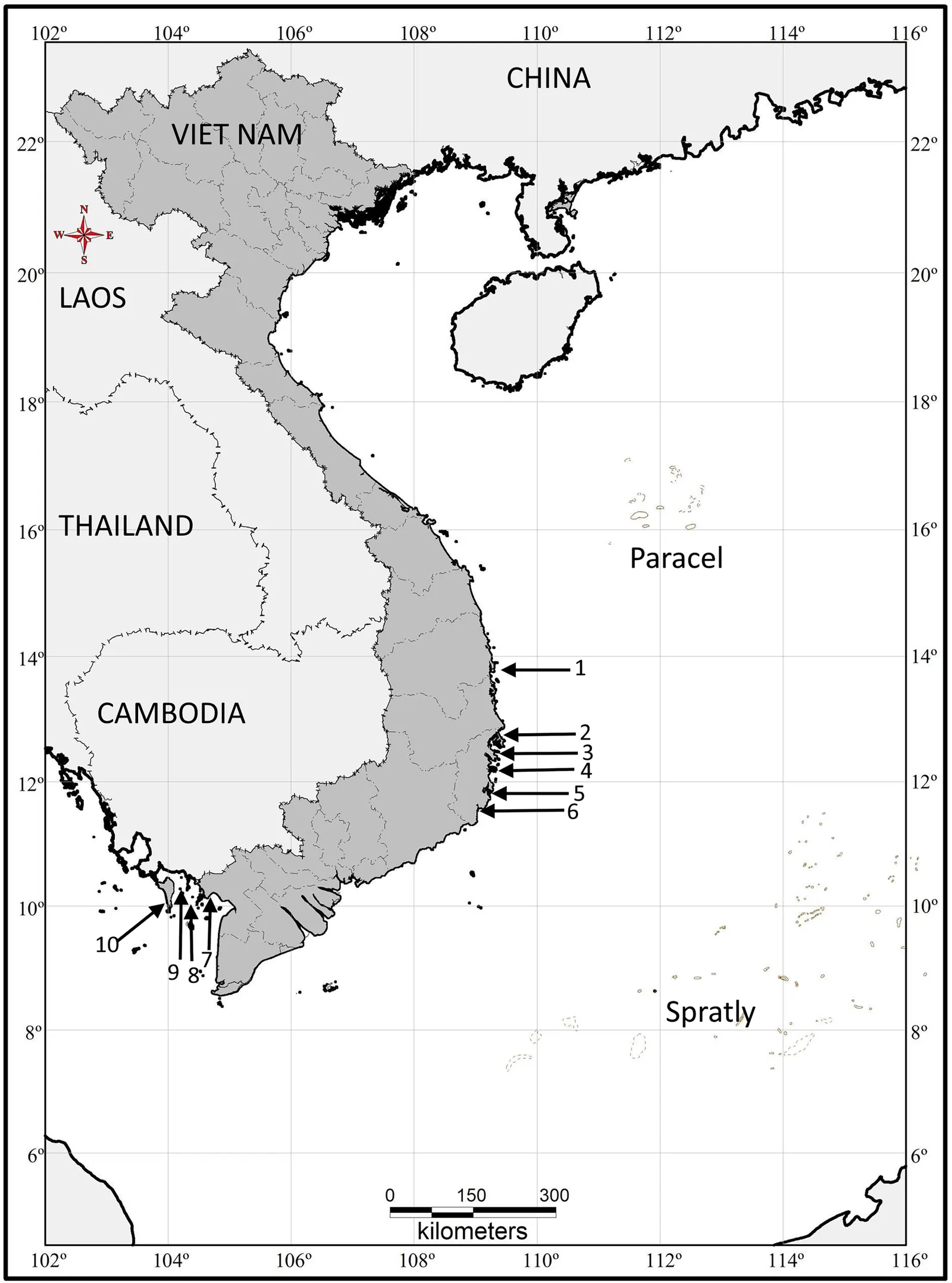
Map of Vietnam and ten sampling sites (arrow). 1 = Cu Mong Lagoon, 2 = Xuan Tu, 3 = Hon Khoi, 4 = Nha Trang, 5 = Cam Ranh Bay, 6 = Ninh Hai, 7 = Kien Luong, 8 = Ba Lua Archipelago, 9 = Hai Tac Archipelago, 10 = Phu Quoc Island. The base map was derived from digital map data provided by the Vietnam Department of Survey, Mapping and Geographic Information (Ministry of Natural Resources and Environment of Vietnam, 2022).

At most study sites, seagrasses occurred on muddy-sand substrates and remained continuously submerged, even during low tide, except at site 4. At this site, *E. acoroides* grew on hard substrates in an open-sea environment. The seagrasses were almost exposed to air during low tide and became fully submerged during high tide. At each site, 15–30 distinct shoots were chosen at random from the beds, with 30–40 meters between sampled shoots. In order to extract DNA at a later time young leaves from each shoot were collected and preserved individually in separate 1.5 mL Eppendorf tubes filled with DESS solution (20% dimethyl sulfoxide, 0.25 M disodium EDTA, and saturated NaCl). In total, 196 samples were collected from ten sampling sites. The coordinates and habitat characteristics of the sampling sites are provided in **Appendix 1**.

### 2.2 DNA extraction, PCR, and microsatellite genotyping

In the laboratory, samples were removed from DESS, rinsed with sterile water, and dried using silica. Dried plant material was separately homogenized for each sample using a bead mill (MM 400, Retsch, Germany) at 22 Hz for 2 min. Approximately 50 mg of finely powdered seagrass tissue was used for genomic DNA extraction with QuickDNA™ Miniprep Plus Kit (Zymo Research, CA, USA) following the manufacturer’s instructions. DNA quality was assessed using agarose gel electrophoresis stained with CSLRunsafe (Cleaver Scientific Ltd, Warwickshire, UK). DNA concentration was quantified using a BioSpec-nano spectrophotometer. In this study, 12 primer pairs were used (**Appendix 2**), which were previously developed by Nakajima et al. (2017). Their physical positions across the *E. acoroides* genome have not been comprehensively mapped. PCR conditions and multiplex PCR also followed the protocol described by Nakajima et al. (2017). Each PCR reaction consisted of 6.25 μl 2X Qiagen Type-IT^®^ (Qiagen, Hilden, Germany), 0.3 μl of each primer, 30–50 ng DNA, and nuclease-free water added to a final volume of 12.5 μL. Amplifications were performed in a PTC-200 Thermo cycler (Biozym-Diagnostik GmbH, Hessisch Oldendorf, Germany).

### 2.3 Fragment analysis and data quality checks

Fragment size analysis was performed at 1^st^BASE (Selangor, Malaysia) using an Applied Biosystems 3730 DNA Analyzer. Allele sizes were scored using GeneMapper^®^ Software version 5.0 (Applied Biosystems). Samples exhibiting missing data or ambiguous allele were re-amplified for confirmation. Potential microsatellite scoring errors, including stuttering, large allele dropout, and null alleles, were assessed by MicroChecker software, version 2.2.3 (Van Oosterhout et al., 2004). Individuals with amplification success at fewer than 12 loci were excluded.

### 2.4 Clonal and genetic diversity

GenClone version 2.0 (Arnaud-Haond and Belkhir, 2007) was used to multilocus genotypes (*MLG*s) for each population. Clonal diversity (*R*) was calculated as *R* = (*G*–1)/(*n*–1) where *G* represents the number of genotypes and n is the number of ramets. *R* values range from 0 to 1, with higher values denoting lower levels of clonality (Byeon et al., 2025). To evaluate the genetic diversity of the ten populations of *Enhalus acoroides*, several indices, including allelic richness (*A*), expected heterozygosity (*H_E_*) under Hardy-Weinberg equilibrium, and observed heterozygosity (*H_O_*) were calculated using GenAlEx Version 6.5 (Peakall and Smouse, 2012). Allelic richness (*A*) was standardized to the same number of *MLG*s (A_22_) using standArich in R version 2.15.3. Deviation from Hardy-Weinberg proportions was tested using a Markov chain algorithm (Guo and Thompson, 1992) implemented in Arlequin 3.5 (Excoffier and Lischer, 2010). Multilocus tests for Hardy-Weinberg equilibrium (HWE) and linkage disequilibrium (LD) for genotypes among pairs of the 12 loci were performed in Arlequin 3.5. FSTAT v2.9.3.2 (Goudet, 1995) was used to determine the inbreeding coefficient (*F_IS_*) based on the method of Weir and Cockerham (1984).

### 2.5 Population structure and connectivity

GenAlEx v6.5 was used to calculate pairwise *F_ST_* to evaluate the genetic differentiation between sites. Slatkin’s linearized *F_ST_* distance [*F_ST_*/(1 − *F_ST_*)] (Slatkin, 1995) was calculated in Arlequin version 3.5. GenAlEx v6.5 was also used to calculate pairwise *F_ST_* to evaluate the genetic differentiation between sites. The significance of pairwise *F_ST_* values was assessed using 10, 100 permutations. To account for multiple testing, *p*-values from the 45 pairwise comparisons were adjusted using three multiple-testing procedures: Bonferroni, Holm–Bonferroni step-down, and Benjamini–Hochberg false discovery rate (FDR) correction, with α = 0.05. Population relationships were visualized using an unweighted pair-group method with arithmetic mean (UPGMA) dendrogram constructed in TFPGA version 1.3 (Miller, 1997). The genetic structure among the 10 populations was inferred from microsatellite data using an individual-based Bayesian clustering approach, and the analysis was implemented in STRUCTURE v2.3.4 (Pritchard et al., 2000). The admixture model with correlated allele frequencies was chosen and the number of genetic clusters (*k*) was predefined from 1 to 10. For each *k*, 15 independent runs were performed, with 100, 000 burn-in iterations followed by 700, 000 MCMC iterations. The optimal number of clusters was determined using both the log probability of the data, L(K) (Rosenberg et al., 2001), and the ΔK method (Evanno et al., 2005), as implemented in Structure Harvester (Earl and vonHoldt, 2012). Discriminant analysis of principal components (DAPC) was performed in R using the adegenet package to evaluate genetic differentiation among sampling populations based on multilocus microsatellite data. Principal component analysis (PCA) was first used to transform and reduce the dimensionality of the multilocus genotype data, followed by linear discriminant analysis (LDA) to maximize discrimination among predefined groups while minimizing within-group variation. The optimal number of principal components was determined using cross-validation implemented in the xvalDapc function, with 90% of individuals used for training and 30 replicate runs. The optimal number of PCs was selected based on the highest mean successful assignment rate and the lowest root mean squared error (RMSE). DAPC was first performed using the 10 sampling populations as predefined groups to visualize genetic differentiation among sampling locations. Subsequently, *a priori* regional groupings were evaluated using four predefined groups: Site 1; Site 4; Site 2 + Site 3; and Sites 5–10. The number of principal components was independently optimized for the four-group analysis using cross-validation. The final four-group DAPC was performed using 35 PCs and three discriminant axes. Individual assignment accuracy was assessed by comparing DAPC-assigned group membership with the predefined group membership.

Analysis of molecular variance (AMOVA) was conducted to evaluate genetic variation at four hierarchical levels: among groups (*F_CT_*), among populations within groups (*F_SC_*), among individuals within populations (*F_IS_*), and within individuals (*F_IT_*), based on the population grouping inferred from the STRUCTURE analysis. In addition, isolation by distance (IBD) among the populations was tested using the Mantel test in XLStat 2020.3.1. The IBD analysis was performed using two matrices: pairwise Slatkin’s linearized genetic distances [*F_ST_*/(1 − *F_ST_*)] and geographic distances (km). Geographic distances between populations were measured along the coastline between sampling sites using geographic coordinates obtained from the National Oceanic and Atmospheric Administration (NOAA) digital map. Historical migration patterns from donor populations to recipient populations was examined with divMigrate online (Sundqvist et al., 2016), based on *Nm* (Alcala et al., 2014) as a relative value of gene flow. Contemporary migration rates were estimated using BayesAss version 3.0 based on a Bayesian approach for multilocus genotype data (Wilson and Rannala, 2003). Higher values indicate stronger genetic exchange (gene flow) among populations. According to Yu et al. (2018), a population with a high relative genetic contribution value (*C_RT_*) should be prioritized for conservation. It was used in *Enhalus acoroides* in China (Yu et al., 2018) and *Thalassia hemprichii* in Vietnam (Nguyen-Nhat et al., 2024). There are two components to the total contribution rate *C_RT(k)_*. *C_RS_*_(k)_ is the rate of contribution of the k^th^ population due to its own diversity, and C_RD(k)_ is the contribution due to its divergence. *C_RT(k)_* = *C_RS(k_*_)_ + _CRD(k)_. Populations with high priority are recommended for conservation.


$$R_{Sk}=\frac{R_{k}}{n};\;R_{Dk}=\sum_{i}^{R_{k}}\frac{n-n_{i}}{nn_{i}};\;R_{Tk}=R_{Sk}+R_{Dk}=\sum_{i}^{R_{k}}\frac{1}{n_{i}}$$

$$C_{RSk}=\frac{R_{Sk}-{\bar{R}}_{D}}{R_{T}};\;C_{RDk}=\frac{R_{Dk}-{\bar{R}}_{D}}{R_{T}};\;C_{RTk}=\frac{R_{Tk}-{\bar{R}}_{T}}{R_{T}}$$

Where *n* is the number of populations, *R_k_* is the number of alleles of the *k*^th^ population, *n_i_* is the number of populations containing the *i^th^* allele, *R_T_* is the allele richness at species level, *R_T_* is the average allele richness among the *n* population. *R_T_* considers both the genetic variation within a population (*R_S_*) and distinctiveness among populations (*R_D_*).

## 3 Results

### 3.1 Clonal and genetic diversity

Among 196 samples collected from ten populations, 12 microsatellite loci were successfully amplified in 159 individuals. A total of 154 unique *MLG*s were identified. Therefore, the final dataset comprised 154 *MLG*s genotyped at 12 microsatellite loci. These analyses were based on 154 unique MLGs rather than the 159 successfully genotyped individuals. The number of alleles per locus ranged from two to 18. Four loci were highly polymorphic, with a large number of alleles, they are Eaco_054 (18 alleles), Eaco_055 (16 alleles), and Eaco_051 and Eaco_447 (10 alleles each). In contrast, several loci exhibited lower allelic diversity. For example, Eaco_019 possessed only two alleles, Eaco_018, Eaco_002, and Eaco_052 had three alleles. Locus Eaco_019 was monomorphic in almost all populations, except Phu Quoc Island (site 10). Clonality varied among populations with 11 (HT, site 9) –27 (Ba Lua, site 8) genotypes per population. The clonal diversity (*R*) was 0.77–1.0. The standardized allelic richness (*A*_22_) across all loci ranged from 2.37 (CM, site 1) to 3.56 (CR, site 4). Expected heterozygosity (*H_E_*) and observed heterozygosity (*H_O_*) ranged from 0.34 to 0.48 and 0.14–0.34, respectively. Inbreeding coefficient (*F_IS_*) ranged from 0.287 – 0.726 (**Table 1**).

**Table 1:** Genetic diversity of *Enhalus acoroides* populations from 10 sites along the coast of Vietnam.

Sites	Latitude (^0^N)	Longitude (^0^E)	*N*s	*N*_all_	*MLG*s	*R*	*A*	*A*_22_	*P*_A_	*H*_O_ (SD)	*H*_E_ (SD)	*F_IS_*
1	13.39	109.12	15	14	14	1.00	2.42	2.37	0.17	0.19 (0.24)	0.36 (0.30)	0.437
2	12.75	109.35	15	13	13	1.00	3.08	2.98	0.04	0.23 (0.31)	0.41 (0.34)	0.400
3	12.57	109.21	15	15	15	1.00	3.67	3.37	0.19	0.27 (0.35)	0.45 (0.26)	0.430
4	12.16	109.21	16	16	16	1.00	2.67	2.51	0.13	0.21 (0.36)	0.34 (0.34)	0.430
5	11.97	109.21	15	14	14	1.00	3.75	3.56	0.22	0.34 (0.34)	0.48 (0.31)	0.287
6	11.61	109.15	15	13	13	1.00	3.25	3.14	0.07	0.25 (0.32)	0.48 (0.28)	0.432
7	10.18	104.60	30	20	19	0.95	2.75	2.62	0.14	0.16 (0.23)	0.37 (0.27)	0.652
8	10.14	104.50	30	28	27	0.96	2.92	2.58	0.20	0.14 (0.25)	0.39 (0.25)	0.726
9	10.32	104.32	30	14	11	0.77	2.67	2.67	0.10	0.16 (0.25)	0.40 (0.29)	0.558
10	10.25	104.09	15	12	12	1.00	3.08	3.05	0.32	0.2 (0.25)	0.44 (0.25)	0.567

Ns, number of samples collected. *N_all_*, number of individuals successfully amplified at all 12 microsatellite loci. *MLG*, number of multilocus genotypes, *R*, genotypic richness; *A*, allelic richness; *A*_22_, allelic richness standardized to 22 genotypes; *P_A_*, private alleles. *H_O_*, observed heterozygosity. *H_E_*, expected heterozygosity. *F_IS_*, inbreeding coefficient. Site abbreviations are provided in **Figure 1**.

Exact tests of Hardy–Weinberg equilibrium (HWE) were performed for each locus within each sampling population. Of the 120 population–locus combinations, 95 were testable and 25 were monomorphic and therefore not tested (**Appendix 3**). Overall, 70 of the 95 testable combinations (73.7%) showed significant departures from HWE at *P* < 0.05. Significant departures were detected in all 10 sampling populations, ranging from 5 of 7 testable loci in Site 4 to 9 of 10 in Site 8. In parallel with the HWE analysis, pairwise linkage disequilibrium (LD) was evaluated among the microsatellite loci within each sampling population. Only polymorphic loci were considered for pairwise LD testing. Across the 10 sampling populations, 423 informative locus pairs were tested, of which 66 (15.6%) showed significant LD at *P* < 0.05 (**Appendix 3**). The number of testable locus pairs varied from 21 in Site 4, which contained seven polymorphic loci, to 66 in Site 10, where all 12 loci were polymorphic. The number of significant LD pairs also varied among populations, ranging from 0 in Site 4 to 13 in Site 10. Relatively high numbers of significant LD pairs were observed in Site 8 (11 pairs) and Site 6 (9 pairs), whereas fewer significant pairs were detected in Sites 1, 2, 3, 5, 7, and 9 (7, 5, 6, 7, 2, and 6 pairs, respectively). These results indicate that significant non-random associations among loci occurred in several sampling populations, but their frequency varied substantially among populations.

### 3.2 Population structure and connectivity

Pairwise *F_ST_* values among the ten populations ranged from 0.06 to 0.39. The greatest genetic differentiation was observed between Cu Mong Lagoon (site 1) and Nha Trang Bay (site 4) (*F_ST_* = 0.39), whereas the lowest differentiation occurred between Cam Ranh Bay (site 5) and Ninh Hai (site 6) (*F_ST_* = 0.06). All pairwise *F_ST_* and Slatkin’s linearized *F_ST_* comparisons were statistically significant (p < 0.01; **Table 2**). Prior to multiple-testing correction, all 45 pairwise comparisons were significant. Following correction, 41 comparisons remained significant under the Bonferroni procedure, whereas all 45 comparisons remained significant after Holm–Bonferroni and Benjamini–Hochberg corrections. Analysis of molecular variance (AMOVA) based on the two geographic regions (eastern waters and western waters) revealed significant genetic differentiation both between regions (*F_CT_* = 0.04, p < 0.05) and among populations within regions (*F_SC_* = 0.20, p < 0.01) (**Table 3**), indicating significant genetic structure at both hierarchical levels. The UPGMA dendrogram grouped the ten populations into four major clusters: (I) Cu Mong Lagoon (site 1); (II) Nha Trang Bay (site 4); (III) Xuan Tu (site 2) and Hon Khoi (site 3) in Van Phong Bay; (IV) the remaining six populations, comprising Cam Ranh Bay (site 5), Ninh Hai (site 6), and the four populations from the Gulf of Thailand (sites 7–10) (**Figure 2**, lower panel). Bayesian clustering analysis in STRUCTURE identified *k* = 4 as the most likely number of genetic clusters based on the ΔK method. Results for the other tested *k* values are provided in **Appendix 4**. The inferred population structure was consistent with the UPGMA results, separating the ten populations into four genetic groups: site 1, site 4, sites 2–3, and the remaining six sites (**Figure 2**, upper panel). DAPC revealed clear genetic differentiation among the sampling populations. Cross-validation identified 40 principal components as the optimal number of PCs for discriminating the 10 sampling populations, yielding the highest mean successful assignment rate (82.78%) and the lowest RMSE (0.214). The first two discriminant axes accounted for 86.21% of the total discriminant variation, with the first discriminant axis (LD1) accounting for 79.26% and the second axis (LD2) accounting for 6.95%. Site 4 and Site 1 were strongly separated from the remaining populations, whereas Sites 2, 3, and 5–10 showed greater overlap in discriminant space.

**Table 2:** Pairwise genetic differentiation among ten populations of *Enhalus acoroides*.

Sampling sites										
	1	2	3	4	5	6	7	8	9	10
1		0.30	0.45	0.65	0.32	0.38	0.38	0.51	0.49	0.32
2	0.23		0.07	0.34	0.20	0.23	0.24	0.32	0.28	0.21
3	0.31	0.06		0.32	0.19	0.16	0.26	0.37	0.26	0.25
4	0.39	0.25	0.24		0.42	0.43	0.41	0.50	0.42	0.43
5	0.25	0.17	0.16	0.30		0.07	0.24	0.20	0.17	0.14
6	0.28	0.19	0.14	0.30	0.06		0.31	0.28	0.20	0.18
7	0.28	0.19	0.21	0.29	0.20	0.24		0.27	0.21	0.16
8	0.34	0.24	0.27	0.33	0.17	0.22	0.21		0.17	0.27
9	0.33	0.22	0.20	0.30	0.15	0.17	0.17	0.15		0.15
10	0.24	0.18	0.20	0.30	0.12	0.15	0.14	0.21	0.13	

Pairwise *F_ST_* values are shown in the lower left triangle, and Slatkin’s linearized values are shown in the upper right triangle. Site abbreviations are provided in **Figure 1**. All pairwise comparisons were statistically significant based on uncorrected p-values (*p* < 0.05).

**Table 3:** Analysis of molecular variance (AMOVA) for 10 *Enhalus acoroides* populations based on 12 microsatellite loci under two grouping schemes: (1) western and eastern Vietnamese waters, and (2) the four genetic clusters identified by STRUCTURE.

Source of variation	Degrees of freedom	Sum of squares	Variance of components	% variation	Fixation indices	Probability
Western waters (Gulf of Thailand) vs Eastern waters (South China Sea)						
Among groups (*F_CT_*)	1	42.67	0.12	3.79	0.04	<0.05
Among populations within groups (*F_SC_*)	8	173.79	0.60	19.03	0.20	<0.01
Among individuals within populations (*F_IS_*)	144	524.01	1.20	37.79	0.49	<0.01
Within individuals (*F_IT_*)	154	192.00	1.25	39.39	0.61	<0.01
Following four groups of structure						
Among groups (*F_CT_*)	3	115.24	0.38	11.64	0.12	<0.01
Among populations within groups (*F_SC_*)	6	101.23	0.43	13.26	0.15	<0.01
Among individuals within populations (FIS)	144	524.00	1.20	36.77	0.49	<0.01
Within individuals (*F_IT_*)	154	192.00	1.25	38.33	0.62	<0.01

**Figure 2:**
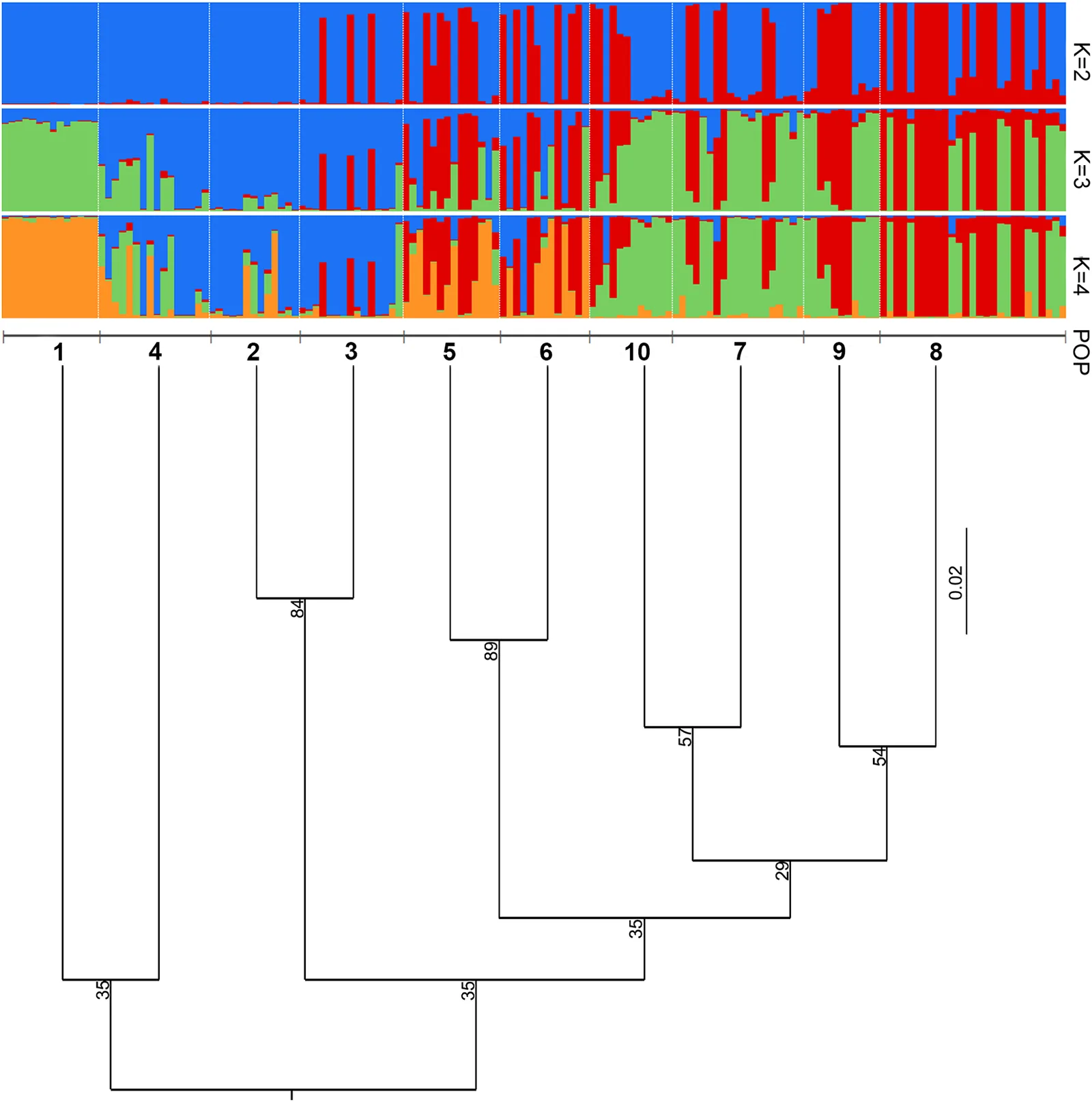
Population genetic structure of *Enhalus acoroides* inferred from 12 microsatellite loci. The upper panel shows the Bayesian clustering analysis performed using STRUCTURE v2.3.4 at *k* = 4, corresponding to the highest ΔK value. Each vertical bar represents an individual, and colors indicate the estimated membership coefficients assigned to the inferred genetic clusters. For each *k*, 15 independent runs were performed, with 100, 000 burn-in iterations followed by 700, 000 Markov chain Monte Carlo (MCMC) iteration. The lower panel shows the UPGMA dendrogram generated in TFPGA v1.3 based on population genetic relationships. Site abbreviations are provided in **Figure 1**.

To further evaluate the proposed regional grouping, DAPC was subsequently performed using four predefined groups: Site 1, Site 4, Site 2 + Site 3, and Sites 5–10. Cross-validation identified 35 PCs as the optimal number of principal components, yielding a mean successful assignment rate of 96.33% and the lowest RMSE (0.0683). The predefined groups showed distinct separation in discriminant space, with Site 1 and Site 4 showing strong separation from the other groups along LD2 and LD1, respectively. The Site 2 + Site 3 group was separated primarily along LD2 from the Sites 5–10 group, although some overlap was observed between these two groups (**Figure 3**). The final four-group DAPC correctly assigned 149 of 154 individuals (97.39%) to their predefined groups. All individuals from Site 1, Site 4, and Sites 5–10 were correctly assigned (100%), whereas 24 of 28 individuals in the Site 2 + Site 3 group were correctly assigned (85.7%); four individuals were assigned to the Sites 5–10 group. Overall, DAPC provided strong support for the proposed grouping of Site 1 and Site 4 and demonstrated high discriminatory performance for the four predefined groups, while indicating some overlap between Site 2 + Site 3 and Sites 5–10. An additional AMOVA based on the four STRUCTURE-defined clusters showed that 11.64% of the total genetic variation occurred among clusters (*F_CT_*, p < 0.05), whereas 13.26% occurred among populations within clusters (*F_SC_*), and 36.77% occurred among individuals within populations (*F_IS_*), and 38.33% occurred within individuals (*F_IT_*) (**Table 3**). A Mantel test detected a significant but relatively weak pattern of isolation by distance (IBD), with a positive correlation between genetic and geographic distances (Mantel *r* = 0.33, p < 0.05; **Figure 4**). This modest correlation indicates that geographic distance may contribute to, but does not fully explain, the observed genetic differentiation among *E. acoroides* populations.

**Figure 3:**
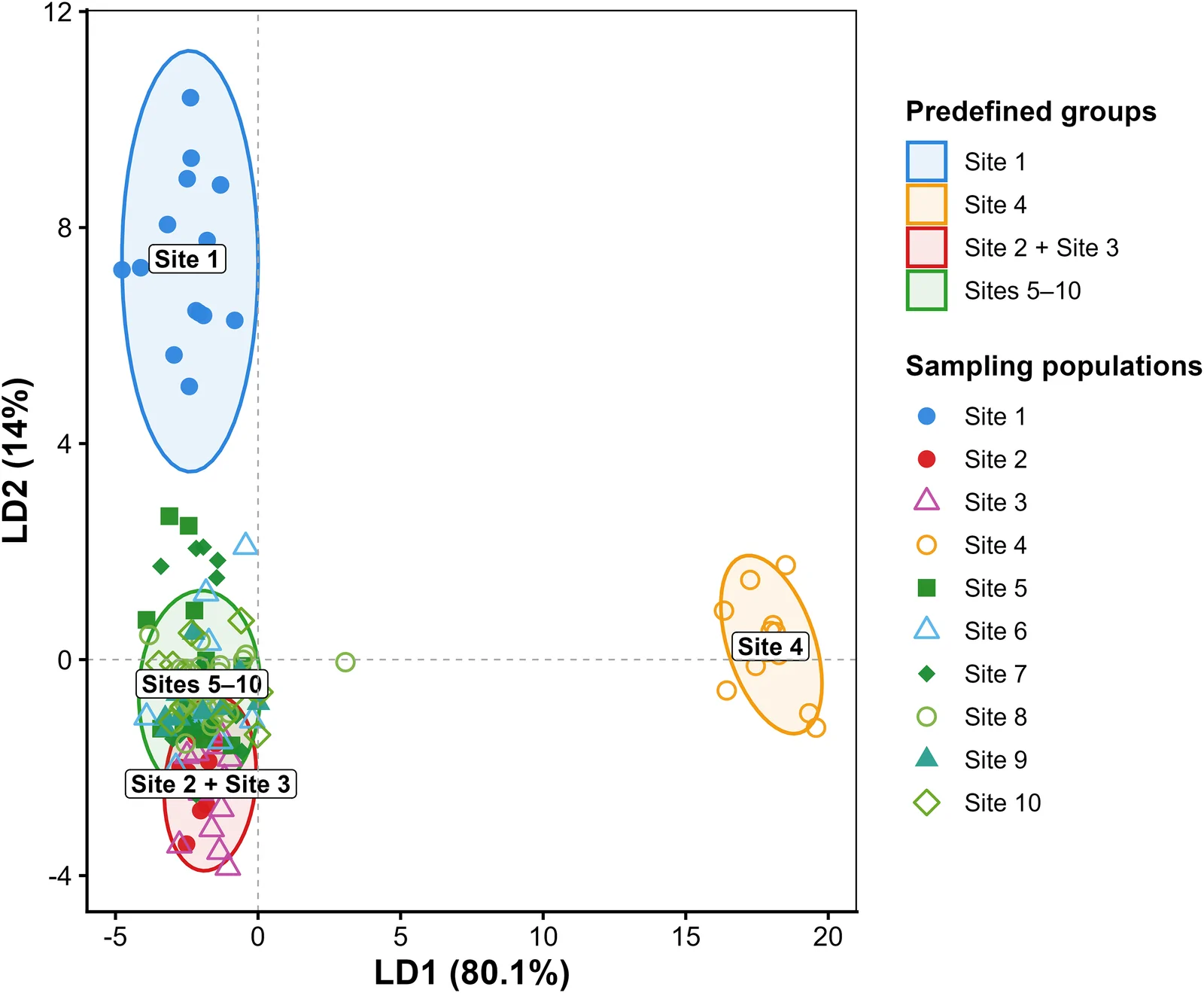
DAPC evaluation of the proposed four-group regional structure based on multilocus microsatellite data. Individual genotypes are shown along the first two discriminant axes (LD1 and LD2), accounting for 80.1% and 14.0% of the total discriminant variation, respectively. Colors and symbols denote the 10 sampling populations, while shaded 95% confidence ellipses indicate the four predefined groups: Site 1, Site 4, Site 2 + Site 3, and Sites 5–10. The analysis was conducted using 35 principal components and three discriminant axes.

**Figure 4:**
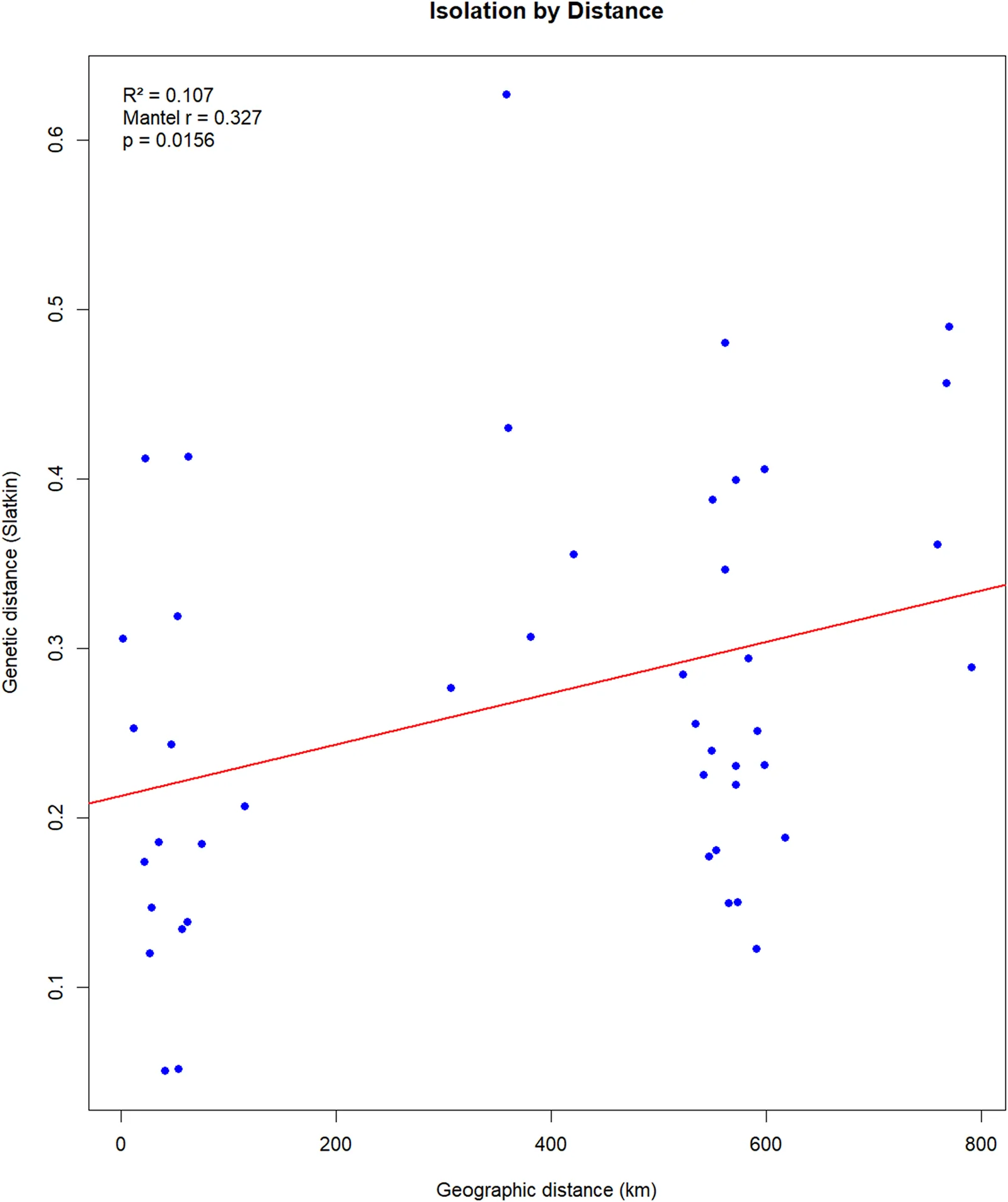
Isolation-by-distance (IBD) analysis of *Enhalus acoroides* populations based on pairwise linearized *F_ST_*/(1–*F_ST_*) plotted against geographic distance (km). The relationship was evaluated using a Mantel test and revealed a significant positive correlation (r = 0.327, p = 0.016; α = 0.05), indicating that genetic differentiation increases with geographic distance. Statistical analyses were performed using XLSTAT v2020.3.1.

Gene flow differed among the ten populations. Overall, gene flow was stronger among populations within the same geographic region than between the Eastern waters and the Western waters. Contemporary migration rates estimated using BayesAss indicated predominantly asymmetric gene flow among several populations. The highest migration rate was inferred from Hon Khoi (site 3) to Xuan Tu (site 2) (*m* = 0.162), followed by migration from Cam Ranh Bay (site 5) to Ninh Hai (site 6) (*m* = 0.115), Hai Tac Archipelago (site 9) to Phu Quoc Island (site 10) (*m* = 0.099), and Hai Tac Archipelago to Ba Lua Archipelago (site 8) (*m* = 0.096). Other relatively high migration rates were observed from Ninh Hai to Xuan Tu (*m* = 0.063), Hon Khoi to Ninh Hai (*m* = 0.054), and Cam Ranh Bay to Xuan Tu (*m* = 0.050) (**Table 4**). In contrast, gene flow from the eastern populations to Nha Trang Bay (site 4) was very limited. Within Eastern waters, migration rates ranged from 0.013 to 0.162, whereas values within Western waters ranged from 0.009 to 0.099. Historical gene flow among *Enhalus acoroides* populations was inferred using divMigrate, revealing heterogeneous and asymmetric patterns of gene exchange among populations (**Figure 5**). Population contribution analysis showed that both the contribution to diversity (*C_RT_*) and contribution to differentiation (*C_RS_*) were positive for populations at Hon Khoi (site 3), Cam Ranh Bay (site 5), Hai Tac Archipelago (site 9), and Phu Quoc Island (site 10), whereas all remaining populations exhibited negative values. Cam Ranh Bay (site 5) contributed the most to overall genetic diversity (2.80%), followed by Hon Khoi (site 3) (2.31%), Hai Tac Archipelago (site 9) (1.64%), and Phu Quoc Island (site 10) (0.29%) (**Figure 6**).

**Table 4:** Contemporary migration rates (*m*) among *Enhalus acoroides* populations estimated using BayesAss.

Sampling sites										
	1	2	3	4	5	6	7	8	9	10
1		0.015	0.014	0.015	0.014	0.014	0.015	0.014	0.014	0.015
2	0.019		0.015	0.015	0.015	0.015	0.018	0.015	0.017	0.017
3	0.013	**0.162**		0.021	0.013	**0.054**	0.015	0.015	0.014	0.014
4	0.013	0.014	0.013		0.013	0.013	0.016	0.014	0.013	0.015
5	**0.044**	**0.050**	0.015	0.022		**0.115**	0.015	0.015	0.015	**0.026**
6	**0.048**	**0.063**	0.014	0.015	0.014		0.015	0.015	0.015	0.017
7	0.012	0.012	0.012	0.014	0.011	0.018		0.012	0.012	0.013
8	0.009	0.010	0.009	0.010	0.009	0.009	0.009		0.009	0.009
9	0.016	0.017	0.016	0.017	0.016	0.022	0.019	**0.096**		**0.099**
10	0.018	0.018	0.016	0.023	0.015	**0.029**	**0.031**	0.027	0.016	

Values represent migration rates from the source populations listed in the rows to the recipient populations listed in the columns. Site abbreviations are provided in **Figure 1**.

Bold values indicate high levels of gene flow between populations.

**Figure 5:**
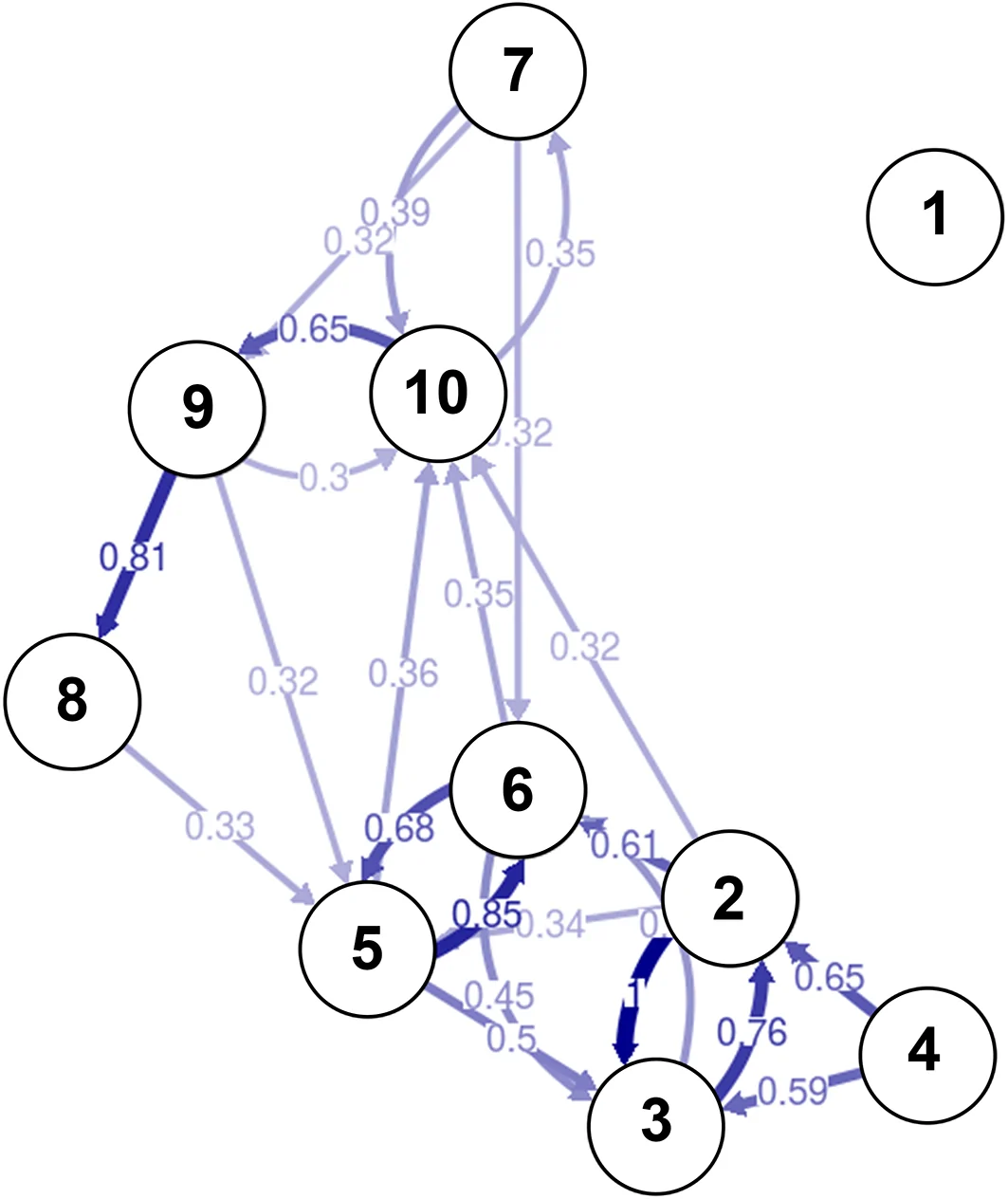
Historical gene flow (*M*) among *Enhalus acoroides* populations inferred using divMigrate online. Circles represent populations, and arrows indicate the direction of historical gene flow. Numbers on the arrows denote the estimated mutation-scaled migration rates (*M*), while arrow thickness is proportional to the magnitude of gene flow. Site abbreviations are provided in **Figure 1**.

**Figure 6:**
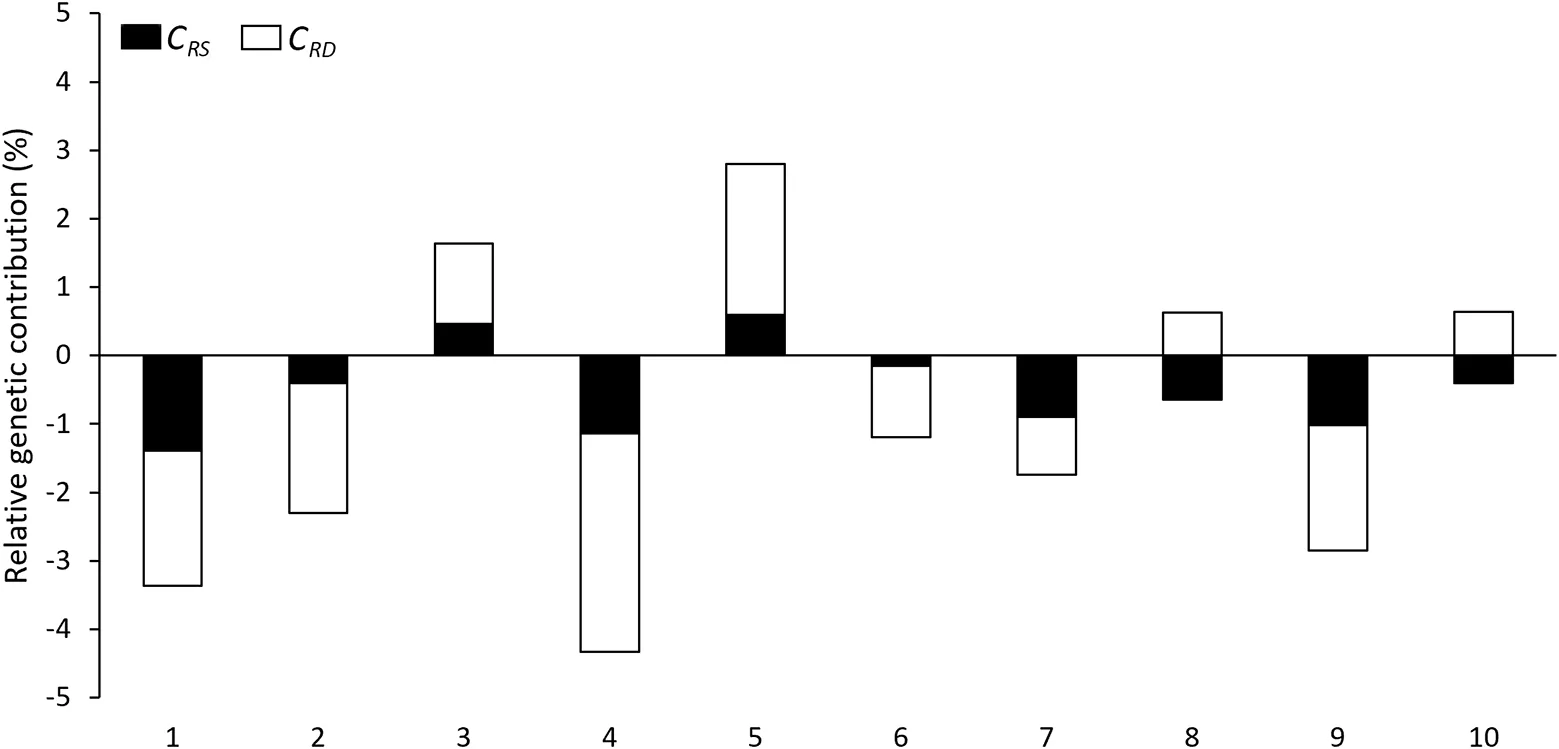
Relative genetic contributions of ten *Enhalus acoroides* populations along the coast of Vietnam. Black bars (*C_RS_*) indicate the contribution of each population to overall genetic diversity, and white bars (*C_RD_*) indicate its contribution to genetic distinctiveness. Site abbreviations are provided in **Figure 1**.

## 4 Discussion

This study provides new insights into the population genetic structure of the dominant tropical seagrass *Enhalus acoroides* in the Eastern and Western waters of Vietnam, and genetic connectivity among populations distributed along the central coast and the An Giang coast. The high clonal diversity observed across populations (R = 0.77–1.00) indicates that *E. acoroides* populations are maintained predominantly through sexual reproduction. Dierick et al. (2021) reported a slightly lower clonal diversity (R = 0.67) for *E. acoroides* in a central Vietnamese lagoon, whereas Nakajima et al. (2014) similarly concluded that sexual reproduction predominates throughout the Indo-West Pacific. Comparable patterns have also been reported for another species in Hydrocharitaceae, the tropical seagrass *Thalassia hemprichii*, in central Vietnam, where high fruit production and seedling recruitment indicate substantial recruitment through sexual reproduction (Nguyen et al., 2022a). Likewise, populations of *T. hemprichii* in the Philippines exhibited similarly high clonal diversity (R = 0.70–1.00) (Arriesgado et al., 2023). In contrast, *Halophila beccarii* populations in Lang Co lagoon, Central Vietnam showed substantially lower clonal diversity (Phan et al., 2017), highlighting marked differences in reproductive strategies among seagrass species. Among the Vietnamese populations, Hon Khoi (site 3) and Cam Ranh Bay (site 5) exhibited the highest standardized allelic richness (A22 = 3.37 and 3.56, respectively). Overall, the level of genetic diversity observed in this study was comparable to that reported for *E. acoroides* populations in central Vietnamese lagoons (Dierick et al., 2021) and in Hainan, China (Yu et al., 2018), but lower than that reported for populations in the Philippines (Nakajima et al., 2017; Arriesgado et al., 2023). The relatively high *F_IS_* values observed in the present study indicate a substantial deficiency of heterozygotes in the sampled populations. Several factors may contribute to this pattern, including null alleles, population subdivision, restricted gene exchange, demographic history, and sampling effects. Thus, the high *F_IS_* values may reflect a combination of biological and demographic processes rather than strong inbreeding alone. In contrast, the Cu Mong Lagoon population exhibited the lowest allelic richness, consistent with previous observations by Dierick et al. (2021). The reduced genetic diversity observed in Cu Mong Lagoon may be related to the documented habitat loss and fragmentation in the lagoon; however, this association should be interpreted cautiously because habitat degradation was not directly assessed in the present study. Historically extensive *E. acoroides* meadows have become highly fragmented, with only approximately 43 ha remaining, representing an 83% decline (approximately 207 ha lost) in seagrass area (Nguyen et al., 2021). Habitat loss and meadow fragmentation are expected to reduce genetic diversity through the loss of genotypes, increased clonality, and restricted gene flow, thereby decreasing the long-term evolutionary potential and resilience of seagrass populations (Procaccini et al., 2007; Litsi-Mizan et al., 2024).

*Enhalus acoroides* meadows along the Vietnamese coast exhibited substantial genetic differentiation among populations. The highest pairwise *F_ST_* values were observed between Cu Mong Lagoon and the remaining populations, particularly Nha Trang Bay. Likewise, Nha Trang Bay showed high levels of genetic differentiation from most other populations. Similar patterns have been reported in central Vietnam, where *E. acoroides* populations exhibited strong genetic differentiation among lagoons but relatively low differentiation, and consequently high genetic connectivity, among meadows within individual lagoons (Dierick et al., 2021). High levels of population differentiation have also been reported for *E. acoroides* in the Philippines (Nakajima et al., 2017), Indonesia (Putra et al., 2018), and China (Yu et al., 2018). The significant but relatively weak isolation-by-distance (IBD) pattern (Mantel r = 0.33, p < 0.05) suggests that geographic distance contributes to genetic differentiation among *E. acoroides* populations, but is unlikely to be the sole factor shaping the observed population structure. The weak strength of this relationship indicates that other factors, including habitat characteristics, restricted gene flow, and oceanographic circulation, may also contribute to the spatial genetic structure observed along the Vietnamese coast. Together, these patterns suggest that geographic distance contributes to population differentiation, while additional ecological and oceanographic factors may further shape the spatial genetic structure of *E. acoroides*. Among the populations examined in the present study, Nha Trang Bay was characterized by relatively low genetic diversity, high genetic differentiation (*F_ST_*), and limited contemporary gene flow from other populations. This pattern may be associated, at least in part, with the distinctive habitat occupied by this population. Unlike the other study sites, where *E. acoroides* occurs mainly on sheltered muddy-sand substrates, the Nha Trang Bay population grows on dead coral and coarse-grained sediments in an exposed open-coast environment (**Figure 7**). These contrasting environmental conditions, including stronger wave action and currents, relatively stable oceanic salinity, and lower organic matter accumulation may affect propagule establishment and potentially contribute to the observed genetic divergence. Although the distinct habitat characteristics of Nha Trang Bay may contribute to the observed genetic differentiation, the present study does not directly test local adaptation. Because the microsatellite markers used here are assumed to be predominantly neutral, the observed genetic structure should primarily be interpreted as evidence of population differentiation and restricted gene flow rather than direct evidence of adaptive divergence.

**Figure 7:**
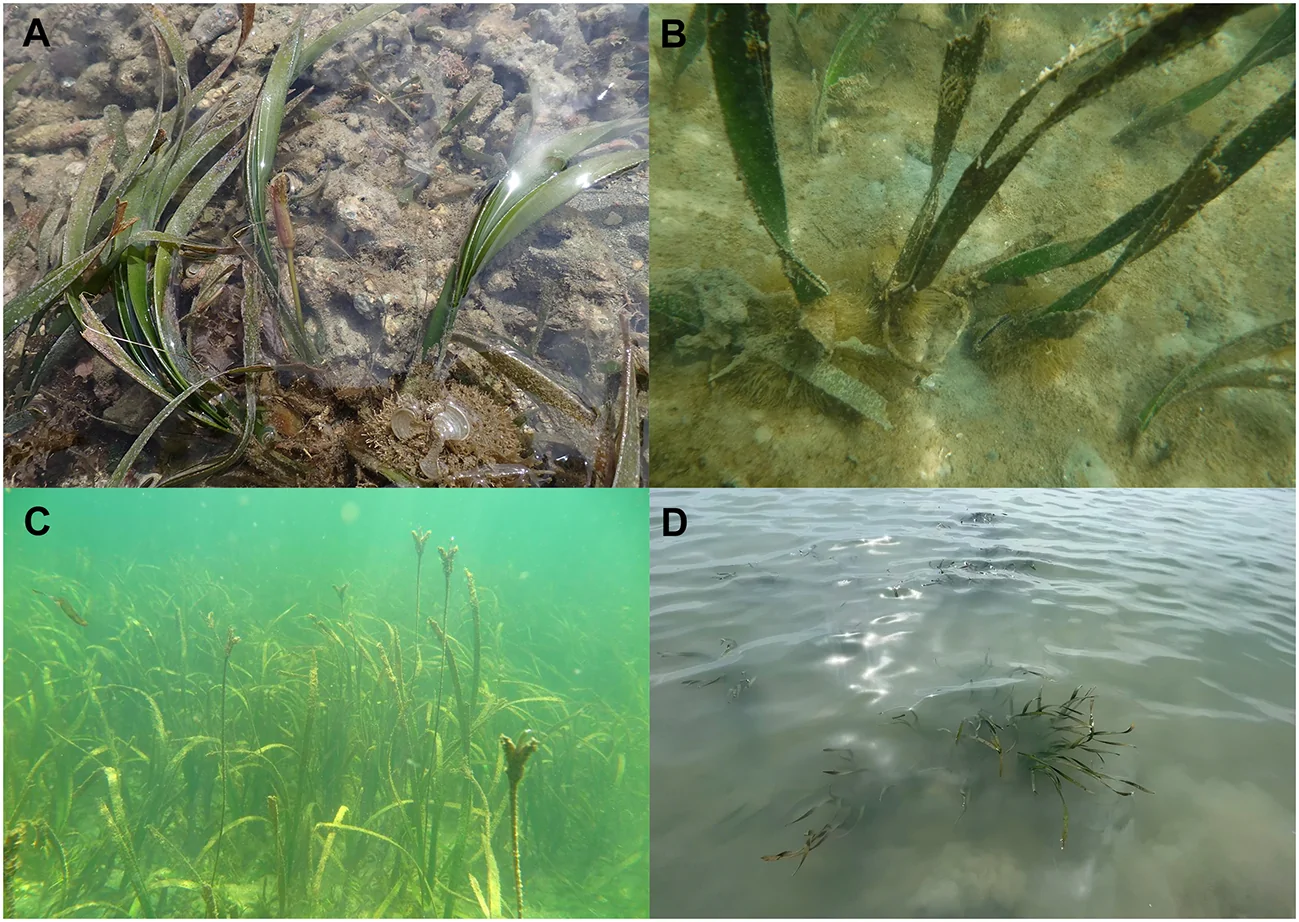
Different substrate types at *Enhalus acoroides* populations. **(A)** Dead coral substrate in Nha Trang Bay. **(B)** Sandy mud substrate in Hon Khoi. **(C)** Sandy mud substrate in Cam Ranh Bay. **(D)** Muddy sand substrate in Cu Mong Lagoon.

The historical gene flow analysis revealed an apparently contrasting pattern at Cu Mong Lagoon (Site 1), where no detectable historical gene flow was observed with geographically neighboring populations, whereas gene flow was inferred between some populations from more distant geographic regions. This pattern suggests that geographic proximity does not necessarily reflect historical connectivity in *E. acoroides*. A similar pattern has been reported along the South-Central Coast of Vietnam, where geographically distant lagoons showed lower genetic differentiation than some geographically neighboring lagoons, suggesting that geographic distance alone does not necessarily predict genetic connectivity (Dierick et al., 2021).

Local habitat discontinuity and historical fragmentation of seagrass meadows may have contributed to restricted connectivity between Cu Mong and nearby populations. In particular, the *E. acoroides* meadow at Cu Mong has experienced substantial degradation, which may have reduced the continuity of suitable habitat and potentially limited connectivity with neighboring populations. However, because the timing and extent of habitat degradation relative to the historical demographic processes cannot be determined from the present data, this interpretation should be considered a plausible explanation rather than a demonstrated causal mechanism. Conversely, historical gene flow between geographically distant populations may reflect past oceanographic connectivity or dispersal events that are not captured by present-day geographic distance. Similar evidence from Hainan showed restricted gene flow among lagoonal populations but connectivity among open-water populations, with geographic isolation and ocean currents proposed as important factors shaping genetic connectivity (Yu et al., 2018). The contrasting patterns may reflect temporal changes in connectivity, with historical and contemporary connectivity being shaped by different combinations of habitat continuity and oceanographic processes.

Experimental evidence supports the potential importance of substrate and hydrodynamic conditions for *E. acoroides* recruitment. Ambo-Rappe (2022) showed that 64% of *E. acoroides* seeds dispersed on fine-sand substrates at a moderately exposed site survived and developed into established seedlings after 40 days. In contrast, seed survival on coarse sand at a highly exposed site and on hard substrates at a moderately exposed site declined rapidly to only 2.0% and 1.4%, respectively, by day 40. Similar patterns have been reported for other seagrass species. Pereda-Briones et al. (2018) demonstrated that the interaction between substrate type and hydrodynamic conditions strongly influences propagule retention and establishment in *Posidonia oceanica*, *Cymodocea nodosa*, and *Zostera marina*. Likewise, field restoration experiments with *Zostera marina* showed that seed burial and protection from hydrodynamic disturbance significantly increased seedling establishment (Unsworth et al., 2024). Together, these findings suggest that the distinctive physical environment of Nha Trang Bay could influence recruitment success and contribute to its demographic and genetic isolation, although this hypothesis requires direct experimental testing. Habitat heterogeneity has also been shown to influence genetic differentiation in other seagrass species. For example, Hernawan et al. (2023) demonstrated that habitat differences contributed to population differentiation and local adaptation in *T. hemprichii*. Similarly, Nguyen et al. (2022a) found that eight populations of *T. hemprichii* from southern Vietnam formed two major genetic groups corresponding to two contrasting habitat types. Environmental variables such as temperature and salinity may further reinforce population divergence. For instance, *Zostera marina* populations on Jeju Island, Korea, possess a distinct genetic composition and are strongly differentiated from mainland Korean populations, a pattern attributed primarily to differences in sea temperature (Byeon et al., 2025). Likewise, Ries et al. (2023) reported that the salinity gradient in the Baltic Sea strongly influenced the genetic diversity and population structure of *Z. marina*, with eastern Swedish populations at low salinity exhibiting extremely low clonal diversity, in some cases consisting of only a few or even a single clone. The asymmetric contemporary migration patterns detected in this study are broadly consistent with regional oceanographic circulation. Gene flow was generally stronger within the South China Sea and within the Gulf of Thailand than between these two marine regions, suggesting that seasonal circulation patterns and relatively weak water exchange through the Gulf of Thailand may limit propagule dispersal between the two regions (Wu et al., 2026). The asymmetric patterns of gene flow may be partly associated with seasonal oceanographic circulation. Fang et al. (2012) reported seasonal reversals in South China Sea currents, which may influence propagule transport and directional connectivity. In our study, stronger migration pathways included Hon Khoi (site 3) to Xuan Tu (site 2), Cam Ranh Bay (site 5) to Ninh Hai (site 6), and Hai Tac Archipelago (site 9) to Ba Lua Archipelago (site 8) and Phu Quoc Island (site 10). These patterns suggest that seasonal circulation may contribute to directional connectivity, although genetic migration reflects cumulative dispersal over multiple generations and cannot be directly attributed to a single seasonal current. Limited exchange between the South China Sea and the An Giang coast of the Gulf of Thailand further suggests that regional circulation and geographic separation may constrain long-distance connectivity. Similar effects of ocean currents on seagrass connectivity have been documented in several Indo-Pacific species (Jahnke et al., 2019; Kendrick et al., 2012; McMahon et al., 2014). Thus, the observed population structure of *E. acoroides* appears to reflect the combined influence of geographic separation, habitat characteristics, and regional oceanographic connectivity.

From a conservation perspective, populations exhibiting high allelic richness, particularly Cam Ranh Bay, Van Phong Bay in the Eastern waters, and Hai Tac Archipelago in the Western waters, may represent candidate populations for conservation and potentially for future restoration programs. Similar conservation priorities have been reported for *E. acoroides* in the Philippines, where weak genetic connectivity and extensive self-recruitment highlighted the importance of conserving multiple interconnected meadows rather than relying solely on a single marine protected area (Nakajima et al., 2017). Yu et al. (2018) identified *E. acoroides* populations from Tielugang and Gangdong, Hainan Island, China, as priority populations for conservation based on their high contributions to regional genetic diversity despite being exposed to substantial anthropogenic disturbance, emphasizing that conservation priorities should consider both genetic diversity and population vulnerability. Their study also demonstrated restricted gene flow among lagoon populations but greater connectivity among open-coast populations, suggesting that geographic isolation and ocean currents jointly shape population structure. Likewise, a microsatellite study of *T. hemprichii* in the Philippines revealed strong population genetic structure and limited contemporary gene flow, supporting the management of individual populations as separate conservation units (Arriesgado et al., 2023). Seascape genetic analyses of *Z. marina* in Skagerrak–Kattegat, eastern North Sea also demonstrated that conservation planning should integrate genetic diversity, population connectivity, and oceanographic processes to maintain regional genetic resilience (Jahnke et al., 2018). Similarly, Arriesgado et al. (2015) showed that although central populations of *Cymodocea serrulata* harbored higher allelic richness and genetic diversity, peripheral populations with lower diversity remained important because of their distinct genetic composition and potential local adaptation. These findings support our recommendation that Cam Ranh Bay, Van Phong Bay, and Hai Tac may represent candidate populations for conservation and potentially for future restoration programs because of their relatively high allelic richness and substantial contributions to genetic diversity (**Figure 6**). However, genetic diversity alone is insufficient to designate candidate populations. The suitability of these populations as donor sources should also be evaluated based on population size and demographic stability, reproductive capacity, habitat condition, genetic compatibility, geographic proximity, and environmental similarity between donor and recipient sites. The genetically distinct Nha Trang Bay population warrant separate conservation consideration to preserve its distinct genetic composition. Although the present study cannot directly assess its adaptive significance, maintaining such genetically differentiated populations may help conserve the breadth of genetic variation within the species. These genetically diverse populations may also represent valuable candidate donor materials for future restoration efforts, as restoration success is enhanced when transplants or seeds are obtained from genetically diverse populations and restoration practices maintain this diversity (Reynolds et al., 2012). However, restoration decisions should integrate genetic information with ecological and demographic assessments of both donor and recipient populations. Recent seagrass restoration frameworks have increasingly emphasized the importance of incorporating genetic information into restoration planning, including consideration of genetic diversity and the selection of appropriate donor populations to enhance the resilience and long-term persistence of restored meadows (Sinclair et al., 2021). In contrast, the Cu Mong Lagoon population may warrant priority attention for active restoration because it exhibited the lowest genetic diversity and limited historical gene flow among the studied populations (**Table 1****;** **Figure 5**). These findings are consistent with previous reports of high anthropogenic disturbance and population isolation of *E. acoroides* in Cu Mong Lagoon (Dierick et al., 2021), as well as the documented substantial decline in seagrass cover in this lagoon (Nguyen et al., 2021). Together, these results indicate that Cu Mong Lagoon as a site of particular conservation concern where genetically informed restoration could be considered, provided that suitable donor populations and recipient-site conditions are identified through additional ecological and demographic assessment.

Several limitations should be considered when interpreting the present findings. First, although the microsatellite markers used in this study were informative, the relatively small number of loci may limit the resolution of population genetic structure and connectivity. Second, the relatively high *F_IS_* values observed in several populations may reflect factors such as inbreeding, population subdivision, or other deviations from Hardy–Weinberg equilibrium, and therefore should be interpreted cautiously. Third, this study did not include direct tests of local adaptation, such as genotype–environment association analyses. Consequently, the observed genetic differentiation cannot be attributed specifically to adaptive divergence. Fourth, an important limitation of the present study is the geographic coverage of the Gulf of Thailand component. Our sampling represents the An Giang coast of the Gulf of Thailand, including Ba Lua Archipelago, Hai Tac Archipelago, and Phu Quoc Island, rather than the Gulf of Thailand as a whole. Consequently, the observed genetic structure and connectivity patterns should be interpreted primarily in the context of populations along the Vietnamese An Giang coast. Connectivity between these populations and *E. acoroides* meadows along the Thai, Cambodian, and Malaysian coasts remains untested. Future studies integrating a larger number of genomic markers with environmental data and direct tests of local adaptation would provide stronger evidence for disentangling neutral population structure from adaptive differentiation. In addition, broader sampling across the Gulf of Thailand, combined with genetic and oceanographic modelling, would be necessary to determine whether the patterns observed here extend across the wider region.

## 5 Conclusion

Overall, *E. acoroides* populations along the Vietnamese coast exhibited significant genetic differentiation and limited genetic connectivity between the Eastern waters and Western waters, together with a significant but relatively weak pattern of isolation by distance. Despite predominantly sexual reproduction and relatively high clonal diversity, substantial variation in genetic diversity was observed among populations, with the Cu Mong Lagoon population exhibiting the lowest allelic richness. This pattern may reflect a combination of demographic and evolutionary processes, with the documented habitat loss and fragmentation in Cu Mong Lagoon representing a possible contributing factor, although habitat degradation was not directly assessed in this study. In contrast, populations from Van Phong Bay, Cam Ranh Bay, and Hai Tac Archipelago exhibited relatively high allelic richness and made relatively high contributions to overall genetic diversity, and may therefore represent candidate populations for conservation and potentially for future restoration programs. The genetically distinct Nha Trang Bay population also warrants separate conservation consideration to preserve its distinctive genetic composition. Overall, these findings provide new insights into the population genetic structure and connectivity of *E. acoroides* across Vietnamese waters and provide a scientific basis for informing conservation, restoration, and sustainable management of seagrass ecosystems along the Vietnamese coast.

## Data Availability

The microsatellite genotype data generated and analyzed in this study are available in Zenodo under record number 22909099 (DOI: 10.5281/zenodo.22909099): https://doi.org/10.5281/zenodo.22909099. All other data supporting the findings of this study are included in the article and its **Supplementary Materials**.
