# Supplementary material for: Population genetic structure and connectivity of Enhalus acoroides along the Southern Vietnamese coastline
Source: Front Plant Sci. 2026 Oct 1;17:1953986. doi: 10.3389/fpls.2026.1953986 (PMC13669108; doi:10.3389/fpls.2026.1953986)
Supplement: Supplementary file 1 [file Table1.docx]

**Appendix 1**: Geographic coordinates, sampling locations, and substratum types of the ten *Enhalus acoroides* populations along the coast of Viet Nam.

| **Sites** | **Name** | **Province** | **Regions** | **Latitude (^0^N)** | **Longitude (^0^E)** | **Substratum** |
| --- | --- | --- | --- | --- | --- | --- |
| 1 | Cu Mong | Dak-Lak | Eastern waters (South China Sea) | 13.39 | 109.12 | Muddy sand |
| 2 | Xuan Tu | Khanh Hoa |  | 12.75 | 109.35 | Sandy mud |
| 3 | Hon Khoi | Khanh Hoa |  | 12.57 | 109.21 | Sandy mud |
| 4 | Nha Trang | Khanh Hoa |  | 12.16 | 109.21 | Dead corals |
| 5 | Cam Ranh | Khanh Hoa |  | 11.97 | 109.21 | Sandy mud |
| 6 | Ninh Hai | Khanh Hoa |  | 11.61 | 109.15 | Sandy mud |
| 7 | Kien Luong | An Giang | Western waters (The Gulf of Thailand) | 10.18 | 104.60 | Muddy sand |
| 8 | Ba Lua | An Giang |  | 10.14 | 104.50 | Muddy sand |
| 9 | Hai Tac | An Giang |  | 10.32 | 104.32 | Muddy sand |
| 10 | Phu Quoc | An Giang |  | 10.25 | 104.09 | Muddy sand |
