# Supplementary material for: Population genetic structure and connectivity of Enhalus acoroides along the Southern Vietnamese coastline
Source: Front Plant Sci. 2026 Oct 1;17:1953986. doi: 10.3389/fpls.2026.1953986 (PMC13669108; doi:10.3389/fpls.2026.1953986)
Supplement: Supplementary file 2 [file Table2.docx]

**Appendix 2**: Characteristics of the 12 microsatellite loci used in this study, including locus name, repeat motif, primer sequences, fluorescent dye, and multiplex assignment. FAM, 6-carboxyfluorescein; HEX, hexachlorofluorescein; TAMRA, tetramethylrhodamine.

| No | Loci | Motif | Sequences | Direction | Label | Multiplex |
| --- | --- | --- | --- | --- | --- | --- |
| 1 | Eaco  001 | [TG]n | GGCTTGAGTTTGTTTAGAATTCTAG | F |  | A |
|  |  |  | TTACATGTGGAATGCATACAC | R | FAM |  |
| 2 | Eaco  010 | [TG]n | GAGTCATAACATTCATATCC | F |  |  |
|  |  |  | AAATGATGCAAAGGCTTGTAC | R | HEX |  |
| 3 | Eaco  018 | [TG]nTA[TG]n | CTTAGCTGGTGCTTGATTTG | F | TAMRA |  |
|  |  |  | CTACCTTGATAACCTATCAC | R |  |  |
| 4 | Eaco  050 | [TG]nTA[TG]nTATG[TA]n | GAATAAATCAAGTCCCTTGAG | F |  | B |
|  |  |  | CAAATAAGATGTGGCTTAC | R | TAMRA |  |
| 5 | Eaco  051 | [GA]nGTGC[GT]nGC[GT]n | CATACAGATGCATGCATACTC | F | HEX |  |
|  |  |  | CTAAGCGCTACGTGGTACTAG | R |  |  |
| 6 | Eaco  054 | [CT]n | GCTTCTAATTAGCATTTTGGACTTCAG | F |  |  |
|  |  |  | ATTTGGGACGTCCAAAGAG | R | FAM |  |
| 7 | Eaco  055 | [TC]nCG[TG]n | CTTTTGCTCCCAAATTGAATG | F |  |  |
|  |  |  | ATGCTTAGTGCAGCTTGTTC | R | FAM |  |
| 8 | Eaco  002 | (TG)nCG(TG)nTATGT  CTGTA(TG)nN28(TG)n | ACTTCAGCCTTGTATGGAACT | F | FAM | C |
|  |  |  | CTATATGCCATAACCATCCATG | R |  |  |
| 9 | Eaco  019 | [CT]n | AGGTATTCCTTACCACCGTTC | F | HEX |  |
|  |  |  | CACGGAGGTCTTTCGAAGTTG | R |  |  |
| 10 | Eaco  048 | [GT]n | CATAAGATGCGTGATCAAGC | F | HEX |  |
|  |  |  | CATGCTACAACTGATCTTCTG | R |  |  |
| 11 | Eaco  052 | [TG]nTC[TG]nTC[TG]n | CAGGCGCACAACGTATGTAC | F | TAMRA |  |
|  |  |  | GAACCACATCATCAGTGTG | R |  |  |
| 12 | EA  447 | [TG]n[GA]n[TG]n | GGGGAGCTATCTTTGTGC | F | HEX |  |
|  |  |  | GAATCACCCGTTCCAAGA | R |  |  |
