# Supplementary material for: Population genetic structure and connectivity of Enhalus acoroides along the Southern Vietnamese coastline
Source: Front Plant Sci. 2026 Oct 1;17:1953986. doi: 10.3389/fpls.2026.1953986 (PMC13669108; doi:10.3389/fpls.2026.1953986)
Supplement: Supplementary file 3 [file Table3.docx]

**Appendix 3:** Summary of Hardy–Weinberg equilibrium and linkage disequilibrium tests by sampling population. *N*: number of individuals; Testable loci: number of loci subjected to HWE testing; Monomorphic loci: loci not tested because they were monomorphic; Significant HWE tests (P < 0.05): number of population–locus combinations showing significant departures from HWE based on the exact test in Arlequin; Locus pairs tested: number of informative pairwise locus combinations subjected to LD testing, calculated as *k*(*k* − 1)/2, where *k* is the number of polymorphic loci in the population; Significant LD pairs (P < 0.05): number of locus pairs showing significant evidence of linkage disequilibrium based on the likelihood-ratio test with permutation procedure in Arlequin. Locus pairs involving monomorphic loci were not considered testable for LD. Statistical significance was evaluated at *P* < 0.05.

| **A. Hardy-Weinberg equilibrium** | | | | | **B. Linkage disequilibrium** | |
| --- | --- | --- | --- | --- | --- | --- |
| **Population** | ***N*** | **Testable loci** | **Monomorphic loci** | **Significant HWE tests (P < 0.05)** | **Locus pairs tested** | **Significant LD pairs**  **(P < 0.05)** |
| Site 1 | 14 | 8 | 4 | 6 | 28 | 7 |
| Site 2 | 13 | 8 | 4 | 7 | 28 | 5 |
| Site 3 | 15 | 11 | 1 | 6 | 55 | 6 |
| Site 4 | 16 | 7 | 5 | 5 | 21 | 0 |
| Site 5 | 14 | 10 | 2 | 6 | 45 | 7 |
| Site 6 | 13 | 10 | 2 | 8 | 45 | 9 |
| Site 7 | 19 | 10 | 2 | 8 | 45 | 2 |
| Site 8 | 27 | 10 | 2 | 9 | 45 | 11 |
| Site 9 | 11 | 10 | 2 | 7 | 45 | 6 |
| Site 10 | 12 | 11 | 1 | 8 | 66 | 13 |
| **Total** | **154** | **95** | **25** | **70** | **423** | **66** |
