# Supplementary material for: Population genetic structure and connectivity of Enhalus acoroides along the Southern Vietnamese coastline
Source: Front Plant Sci. 2026 Oct 1;17:1953986. doi: 10.3389/fpls.2026.1953986 (PMC13669108; doi:10.3389/fpls.2026.1953986)
Supplement: Supplementary file 4 [file Table4.docx]

**Appendix 3**. Population genetic structure of *Enhalus acoroides* inferred from 12 microsatellite loci. The upper panel shows the Bayesian clustering analysis performed using STRUCTURE v2.3.4 at *k* = 5, 6. Each vertical bar represents an individual, and colors indicate the estimated membership coefficients assigned to the inferred genetic clusters. For each *k*, 15 independent runs were performed, with 100,000 burn-in iterations followed by 700,000 Markov chain Monte Carlo (MCMC) iteration. The lower panel shows the UPGMA dendrogram generated in TFPGA v1.3 based on population genetic relationships. Site abbreviations are provided in **Figure 1**.
